# 3-(4-Fluoro­phenyl­sulfon­yl)-2,5,7-trimethyl-1-benzofuran

**DOI:** 10.1107/S1600536810023834

**Published:** 2010-06-26

**Authors:** Hong Dae Choi, Pil Ja Seo, Byeng Wha Son, Uk Lee

**Affiliations:** a Department of Chemistry, Dongeui University, San 24 Kaya-dong Busanjin-gu, Busan 614-714, Republic of Korea; b Department of Chemistry, Pukyong National University, 599-1 Daeyeon 3-dong, Nam-gu, Busan 608-737, Republic of Korea

## Abstract

In the title compound, C_17_H_15_FO_3_S, the 4-fluoro­phenyl ring makes a dihedral angle of 72.67 (5)° with the benzofuran plane. In the crystal, mol­ecules are linked by weak inter­molecular C—H⋯O hydrogen bonds.

## Related literature

For the pharmacological activity of benzofuran compounds, see: Aslam *et al.* (2006 ▶); Galal *et al.* (2009 ▶); Khan *et al.* (2005 ▶). For natural products with benzofuran rings, see: Akgul & Anil (2003 ▶); Soekamto *et al.* (2003 ▶). For the crystal structures of related 2,5-dimethyl-3-phenyl­sulfonyl-1-benzofuran derivatives, see: Choi *et al.* (2008*a* ▶,*b* ▶).
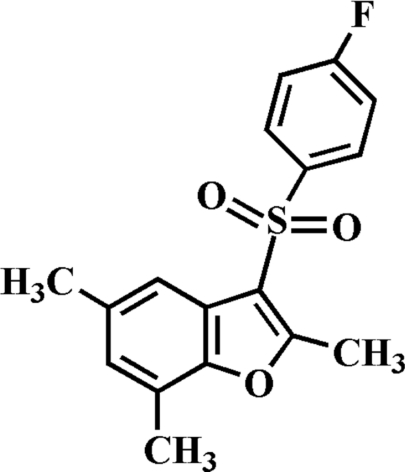


## Experimental

### 

#### Crystal data

C_17_H_15_FO_3_S
*M* *_r_* = 318.35
Triclinic, 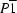

*a* = 8.2851 (2) Å
*b* = 9.3762 (2) Å
*c* = 11.2241 (3) Å
α = 70.441 (1)°
β = 71.177 (1)°
γ = 69.407 (1)°
*V* = 748.07 (3) Å^3^
*Z* = 2
Mo *K*α radiation
μ = 0.24 mm^−1^
*T* = 173 K
0.23 × 0.21 × 0.19 mm

#### Data collection

Bruker SMART APEXII CCD diffractometer
Absorption correction: multi-scan (*SADABS*; Bruker, 2009 ▶) *T*_min_ = 0.948, *T*_max_ = 0.957
13476 measured reflections
3449 independent reflections
3097 reflections with *I* > 2σ(*I*)
*R*_int_ = 0.026

#### Refinement

*R*[*F* ^2^ > 2σ(*F* ^2^)] = 0.038
*wR*(*F* ^2^) = 0.105
*S* = 1.04
3449 reflections
202 parameters
H-atom parameters constrained
Δρ_max_ = 0.28 e Å^−3^
Δρ_min_ = −0.38 e Å^−3^

### 

Data collection: *APEX2* (Bruker, 2009 ▶); cell refinement: *SAINT* (Bruker, 2009 ▶); data reduction: *SAINT*; program(s) used to solve structure: *SHELXS97* (Sheldrick, 2008 ▶); program(s) used to refine structure: *SHELXL97* (Sheldrick, 2008 ▶); molecular graphics: *ORTEP-3* (Farrugia, 1997 ▶) and *DIAMOND* (Brandenburg, 1998 ▶); software used to prepare material for publication: *SHELXL97*.

## Supplementary Material

Crystal structure: contains datablocks global, I. DOI: 10.1107/S1600536810023834/ds2040sup1.cif

Structure factors: contains datablocks I. DOI: 10.1107/S1600536810023834/ds2040Isup2.hkl

Additional supplementary materials:  crystallographic information; 3D view; checkCIF report

## Figures and Tables

**Table 1:** Hydrogen-bond geometry (Å, °)

*D*—H⋯*A*	*D*—H	H⋯*A*	*D*⋯*A*	*D*—H⋯*A*
C10—H10*B*⋯O2^i^	0.98	2.56	3.363 (2)	139
C13—H13⋯O2^ii^	0.95	2.45	3.365 (2)	160
C17—H17⋯O3^iii^	0.95	2.50	3.198 (2)	130

Symmetry codes: (i) 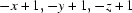; (ii) 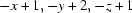; (iii) 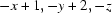.

**Fig. 1.**
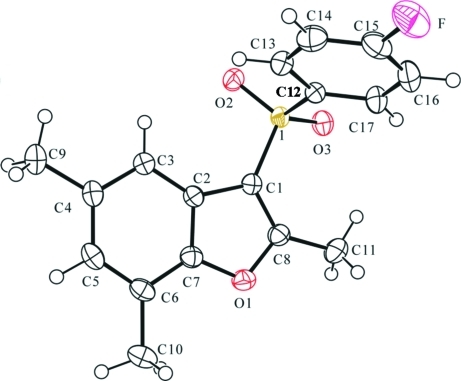
The molecular structure of the title compound with the atom numbering scheme. Displacement ellipsoids are drawn at the 50% probability level. H atoms are presented as a small spheres of arbitrary radius.

**Fig. 2.**
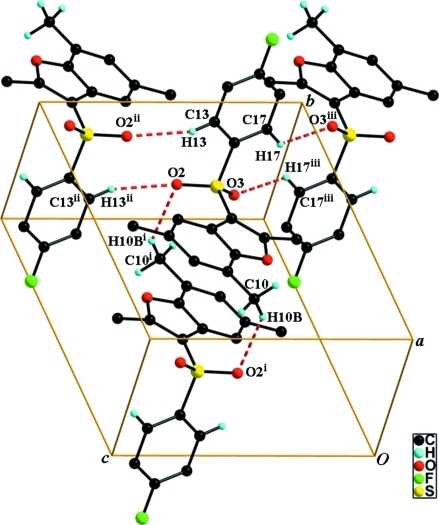
C—H···O interactions (dotted lines) in the crystal structure of the title compound. [Symmetry codes: (i) - x + 1, - y + 1, - z + 1; (ii) - x + 1, - y + 2, - z + 1; (iii) - x + 1, - y + 2, - z.]

## supplementary crystallographic information

### Comment

Compounds containing a benzofuran moiety show potent pharmacological properties such as antifungal (Aslam *et al.*., 2006), antitumor and antiviral (Galal  *et al.*., 2009), antimicrobial (Khan  *et al.*., 2005) activities. These compounds widely occur in nature (Akgul & Anil, 2003; Soekamto  *et al.*. 2003). As a part of our ongoing studies of the effect of side chain substituents on the solid state structures of 2,5-dimethyl-3-phenylsulfonyl-1-benzofuran analogues (Choi  *et al.*., 2008*a, b*), we report the crystal structure of the title compound (Fig. 1).

The benzofuran unit is essentially planar, with a mean deviation of 0.009 (1) Å from the least-squares plane defined by the nine constituent atoms. The 4-fluorophenyl ring makes a dihedral angle of 72.67 (5)° with the benzofuran plane. The crystal packing (Fig. 2) is stabilized by three intermolecular C—H···O hydrogen bonds; the first one between the methyl H atom and the oxygen of the O═S═O unit, with a C10—H10B···O2^i^, the second one between the 4-fluorophenyl H atom and the oxygen of the O═S═O unit, with a C13—H13···O2^ii^, and the third one between the 4-fluorophenyl H atom and the oxygen of the O═S═O unit, with a C17—H17···O3^iii^, respectively (Table 1).

### Experimental

77% 3-Chloroperoxybenzoic acid (538 mg, 2.4 mmol) was added in small portions to a stirred solution of 3-(4-fluorophenylsulfanyl)-2,5,7-trimethy-1-benzofuran (343 mg, 1.2 mmol) in dichloromethane (50 mL) at 273 K. After being stirred at room temperature for 8h, the mixture was washed with saturated sodium bicarbonate solution and the organic layer was separated, dried over magnesium sulfate, filtered and concentrated at reduced pressure. The residue was purified by column chromatography (silica gel, hexane-ethyl acetate, 2:1 v/v) to afford the title compound as a colorless solid [yield 79%, m.p. 414-415 K; *R*_f_ = 0.41 (benzene)]. Single crystals suitable for X-ray diffraction were prepared by slow evaporation of a solution of the title compound in diisopropyl ether at room temperature.

### Refinement

All H atoms were positioned geometrically and refined using a riding model, with C—H = 0.95 Å for aryl and 0.98 Å for methyl H atoms, *U*_iso_(H) = 1.2 *U*_eq_(C)  for aryl and 1.5*U*_eq_(C)  for methyl H atoms.

### Crystal data

**Table:**

C_17_H_15_FO_3_S	*Z* = 2
*M**_r_* = 318.35	*F*(000) = 332
Triclinic, *P*1	*D*_x_ = 1.413 Mg m^−^^3^
Hall symbol: -P 1	Mo *K*α radiation, λ = 0.71069 Å
*a* = 8.2851 (2) Å	Cell parameters from 7810 reflections
*b* = 9.3762 (2) Å	θ = 2.4–27.5°
*c* = 11.2241 (3) Å	µ = 0.24 mm^−^^1^
α = 70.441 (1)°	*T* = 173 K
β = 71.177 (1)°	Block, colourless
γ = 69.407 (1)°	0.23 × 0.21 × 0.19 mm
*V* = 748.07 (3) Å^3^	

### Data collection

**Table:**

Bruker SMART APEXII CCD diffractometer	3449 independent reflections
Radiation source: rotating anode	3097 reflections with *I* > 2σ(*I*)
graphite multilayer	*R*_int_ = 0.026
Detector resolution: 10.0 pixels mm^-1^	θ_max_ = 27.6°, θ_min_ = 2.0°
φ and ω scans	*h* = −10→10
Absorption correction: multi-scan (*SADABS*; Bruker, 2009)	*k* = −12→12
*T*_min_ = 0.948, *T*_max_ = 0.957	*l* = −14→14
13476 measured reflections	

### Refinement

**Table:**

Refinement on *F*^2^	Primary atom site location: structure-invariant direct methods
Least-squares matrix: full	Secondary atom site location: difference Fourier map
*R*[*F*^2^ > 2σ(*F*^2^)] = 0.038	Hydrogen site location: difference Fourier map
*wR*(*F*^2^) = 0.105	H-atom parameters constrained
*S* = 1.04	*w* = 1/[σ^2^(*F*_o_^2^) + (0.0536*P*)^2^ + 0.3586*P*] where *P* = (*F*_o_^2^ + 2*F*_c_^2^)/3
3449 reflections	(Δ/σ)_max_ < 0.001
202 parameters	Δρ_max_ = 0.28 e Å^−^^3^
0 restraints	Δρ_min_ = −0.38 e Å^−^^3^

### Special details

**Table:**

Geometry. All esds (except the esd in the dihedral angle between two l.s. planes) are estimated using the full covariance matrix. The cell esds are taken into account individually in the estimation of esds in distances, angles and torsion angles; correlations between esds in cell parameters are only used when they are defined by crystal symmetry. An approximate (isotropic) treatment of cell esds is used for estimating esds involving l.s. planes.
Refinement. Refinement of F^2^ against ALL reflections. The weighted R-factor wR and goodness of fit S are based on F^2^, conventional R-factors R are based on F, with F set to zero for negative F^2^. The threshold expression of F^2^ > 2sigma(F^2^) is used only for calculating R-factors(gt) etc. and is not relevant to the choice of reflections for refinement. R-factors based on F^2^ are statistically about twice as large as those based on F, and R- factors based on ALL data will be even larger.

### Fractional atomic coordinates and isotropic or equivalent isotropic displacement parameters (Å^2^)

**Table:**

	*x*	*y*	*z*	*U*_iso_*/*U*_eq_	
S	0.48710 (5)	0.89687 (4)	0.29416 (3)	0.02449 (12)	
F	0.98555 (17)	1.27225 (14)	−0.00227 (13)	0.0583 (3)	
O1	0.75715 (15)	0.45344 (12)	0.32852 (11)	0.0300 (2)	
O2	0.39991 (14)	0.94794 (13)	0.41216 (10)	0.0304 (2)	
O3	0.38323 (15)	0.91207 (14)	0.20728 (11)	0.0328 (3)	
C1	0.60789 (19)	0.70192 (17)	0.33737 (14)	0.0254 (3)	
C2	0.67369 (19)	0.62533 (16)	0.45489 (14)	0.0242 (3)	
C3	0.6664 (2)	0.66868 (17)	0.56438 (14)	0.0271 (3)	
H3	0.6038	0.7718	0.5750	0.033*	
C4	0.7533 (2)	0.55674 (19)	0.65748 (15)	0.0299 (3)	
C5	0.8449 (2)	0.40466 (18)	0.63998 (15)	0.0315 (3)	
H5	0.9044	0.3309	0.7046	0.038*	
C6	0.8531 (2)	0.35680 (17)	0.53365 (15)	0.0292 (3)	
C7	0.76463 (19)	0.47267 (17)	0.44329 (14)	0.0262 (3)	
C8	0.6618 (2)	0.59417 (18)	0.26597 (15)	0.0278 (3)	
C9	0.7515 (3)	0.5984 (2)	0.77707 (17)	0.0415 (4)	
H9A	0.7024	0.7123	0.7667	0.062*	
H9B	0.8728	0.5666	0.7883	0.062*	
H9C	0.6782	0.5434	0.8537	0.062*	
C10	0.9501 (2)	0.19388 (19)	0.51553 (18)	0.0373 (4)	
H10A	1.0603	0.1967	0.4485	0.056*	
H10B	0.8753	0.1551	0.4886	0.056*	
H10C	0.9779	0.1236	0.5977	0.056*	
C11	0.6407 (3)	0.5985 (2)	0.13829 (17)	0.0382 (4)	
H11A	0.7576	0.5774	0.0786	0.057*	
H11B	0.5679	0.7027	0.1022	0.057*	
H11C	0.5829	0.5183	0.1498	0.057*	
C12	0.64455 (19)	1.00421 (17)	0.20516 (14)	0.0251 (3)	
C13	0.7224 (2)	1.05360 (19)	0.27106 (15)	0.0309 (3)	
H13	0.6959	1.0257	0.3634	0.037*	
C14	0.8396 (2)	1.1442 (2)	0.20033 (18)	0.0381 (4)	
H14	0.8957	1.1787	0.2429	0.046*	
C15	0.8722 (2)	1.1827 (2)	0.06712 (18)	0.0386 (4)	
C16	0.7969 (2)	1.1347 (2)	−0.00004 (17)	0.0400 (4)	
H16	0.8233	1.1638	−0.0924	0.048*	
C17	0.6816 (2)	1.0430 (2)	0.07044 (15)	0.0333 (3)	
H17	0.6283	1.0068	0.0270	0.040*	

### Atomic displacement parameters (Å^2^)

**Table:**

	*U*^11^	*U*^22^	*U*^33^	*U*^12^	*U*^13^	*U*^23^
S	0.02610 (19)	0.02512 (19)	0.02153 (19)	−0.00398 (14)	−0.00822 (13)	−0.00554 (13)
F	0.0579 (7)	0.0490 (7)	0.0633 (8)	−0.0316 (6)	−0.0054 (6)	0.0012 (6)
O1	0.0343 (6)	0.0252 (5)	0.0327 (6)	−0.0066 (4)	−0.0092 (5)	−0.0100 (4)
O2	0.0309 (6)	0.0314 (6)	0.0254 (5)	−0.0035 (4)	−0.0054 (4)	−0.0092 (4)
O3	0.0340 (6)	0.0364 (6)	0.0301 (6)	−0.0077 (5)	−0.0148 (5)	−0.0058 (5)
C1	0.0276 (7)	0.0249 (7)	0.0237 (7)	−0.0065 (6)	−0.0074 (6)	−0.0056 (5)
C2	0.0247 (7)	0.0234 (7)	0.0237 (7)	−0.0078 (5)	−0.0057 (5)	−0.0034 (5)
C3	0.0296 (7)	0.0255 (7)	0.0252 (7)	−0.0068 (6)	−0.0066 (6)	−0.0055 (6)
C4	0.0329 (8)	0.0324 (8)	0.0235 (7)	−0.0116 (6)	−0.0075 (6)	−0.0022 (6)
C5	0.0325 (8)	0.0285 (8)	0.0289 (8)	−0.0109 (6)	−0.0100 (6)	0.0040 (6)
C6	0.0277 (7)	0.0221 (7)	0.0346 (8)	−0.0089 (6)	−0.0072 (6)	−0.0011 (6)
C7	0.0269 (7)	0.0246 (7)	0.0278 (7)	−0.0097 (6)	−0.0052 (6)	−0.0057 (6)
C8	0.0292 (7)	0.0273 (7)	0.0286 (7)	−0.0079 (6)	−0.0078 (6)	−0.0080 (6)
C9	0.0522 (11)	0.0445 (10)	0.0285 (8)	−0.0108 (8)	−0.0172 (8)	−0.0051 (7)
C10	0.0373 (9)	0.0231 (7)	0.0476 (10)	−0.0058 (6)	−0.0124 (7)	−0.0041 (7)
C11	0.0475 (10)	0.0396 (9)	0.0335 (9)	−0.0092 (8)	−0.0130 (7)	−0.0157 (7)
C12	0.0280 (7)	0.0223 (7)	0.0228 (7)	−0.0032 (5)	−0.0076 (5)	−0.0050 (5)
C13	0.0356 (8)	0.0306 (8)	0.0267 (7)	−0.0066 (6)	−0.0091 (6)	−0.0084 (6)
C14	0.0407 (9)	0.0346 (9)	0.0448 (10)	−0.0121 (7)	−0.0116 (8)	−0.0137 (7)
C15	0.0380 (9)	0.0279 (8)	0.0437 (10)	−0.0123 (7)	−0.0055 (7)	−0.0015 (7)
C16	0.0442 (10)	0.0428 (10)	0.0263 (8)	−0.0140 (8)	−0.0071 (7)	0.0010 (7)
C17	0.0381 (8)	0.0368 (8)	0.0248 (7)	−0.0100 (7)	−0.0109 (6)	−0.0042 (6)

### Geometric parameters (Å, °)

**Table:**

S—O2	1.4384 (11)	C9—H9A	0.9800
S—O3	1.4402 (11)	C9—H9B	0.9800
S—C1	1.7351 (15)	C9—H9C	0.9800
S—C12	1.7669 (15)	C10—H10A	0.9800
F—C15	1.3561 (19)	C10—H10B	0.9800
O1—C8	1.3647 (19)	C10—H10C	0.9800
O1—C7	1.3823 (18)	C11—H11A	0.9800
C1—C8	1.362 (2)	C11—H11B	0.9800
C1—C2	1.452 (2)	C11—H11C	0.9800
C2—C7	1.392 (2)	C12—C17	1.387 (2)
C2—C3	1.396 (2)	C12—C13	1.388 (2)
C3—C4	1.390 (2)	C13—C14	1.387 (2)
C3—H3	0.9500	C13—H13	0.9500
C4—C5	1.406 (2)	C14—C15	1.373 (3)
C4—C9	1.513 (2)	C14—H14	0.9500
C5—C6	1.384 (2)	C15—C16	1.374 (3)
C5—H5	0.9500	C16—C17	1.383 (2)
C6—C7	1.389 (2)	C16—H16	0.9500
C6—C10	1.503 (2)	C17—H17	0.9500
C8—C11	1.484 (2)		
			
O2—S—O3	119.38 (7)	C4—C9—H9C	109.5
O2—S—C1	107.27 (7)	H9A—C9—H9C	109.5
O3—S—C1	109.18 (7)	H9B—C9—H9C	109.5
O2—S—C12	107.00 (7)	C6—C10—H10A	109.5
O3—S—C12	107.22 (7)	C6—C10—H10B	109.5
C1—S—C12	106.04 (7)	H10A—C10—H10B	109.5
C8—O1—C7	107.03 (11)	C6—C10—H10C	109.5
C8—C1—C2	107.63 (13)	H10A—C10—H10C	109.5
C8—C1—S	126.31 (12)	H10B—C10—H10C	109.5
C2—C1—S	126.04 (11)	C8—C11—H11A	109.5
C7—C2—C3	119.44 (13)	C8—C11—H11B	109.5
C7—C2—C1	104.30 (13)	H11A—C11—H11B	109.5
C3—C2—C1	136.26 (14)	C8—C11—H11C	109.5
C4—C3—C2	118.13 (14)	H11A—C11—H11C	109.5
C4—C3—H3	120.9	H11B—C11—H11C	109.5
C2—C3—H3	120.9	C17—C12—C13	121.50 (15)
C3—C4—C5	120.01 (14)	C17—C12—S	118.94 (12)
C3—C4—C9	120.21 (15)	C13—C12—S	119.50 (11)
C5—C4—C9	119.77 (15)	C14—C13—C12	119.12 (15)
C6—C5—C4	123.49 (14)	C14—C13—H13	120.4
C6—C5—H5	118.3	C12—C13—H13	120.4
C4—C5—H5	118.3	C15—C14—C13	118.22 (16)
C5—C6—C7	114.38 (14)	C15—C14—H14	120.9
C5—C6—C10	123.44 (15)	C13—C14—H14	120.9
C7—C6—C10	122.18 (15)	F—C15—C14	118.37 (16)
O1—C7—C6	124.84 (14)	F—C15—C16	118.03 (16)
O1—C7—C2	110.61 (13)	C14—C15—C16	123.59 (16)
C6—C7—C2	124.53 (14)	C15—C16—C17	118.18 (16)
C1—C8—O1	110.43 (13)	C15—C16—H16	120.9
C1—C8—C11	134.21 (15)	C17—C16—H16	120.9
O1—C8—C11	115.35 (13)	C16—C17—C12	119.37 (15)
C4—C9—H9A	109.5	C16—C17—H17	120.3
C4—C9—H9B	109.5	C12—C17—H17	120.3
H9A—C9—H9B	109.5		
			
O2—S—C1—C8	157.19 (13)	C1—C2—C7—O1	−0.13 (16)
O3—S—C1—C8	26.50 (16)	C3—C2—C7—C6	−0.8 (2)
C12—S—C1—C8	−88.73 (15)	C1—C2—C7—C6	178.84 (14)
O2—S—C1—C2	−24.51 (15)	C2—C1—C8—O1	0.16 (17)
O3—S—C1—C2	−155.21 (12)	S—C1—C8—O1	178.72 (10)
C12—S—C1—C2	89.57 (13)	C2—C1—C8—C11	−179.06 (17)
C8—C1—C2—C7	−0.02 (16)	S—C1—C8—C11	−0.5 (3)
S—C1—C2—C7	−178.58 (11)	C7—O1—C8—C1	−0.25 (16)
C8—C1—C2—C3	179.48 (16)	C7—O1—C8—C11	179.14 (13)
S—C1—C2—C3	0.9 (3)	O2—S—C12—C17	−147.39 (12)
C7—C2—C3—C4	0.8 (2)	O3—S—C12—C17	−18.20 (14)
C1—C2—C3—C4	−178.62 (15)	C1—S—C12—C17	98.35 (13)
C2—C3—C4—C5	−0.1 (2)	O2—S—C12—C13	29.85 (14)
C2—C3—C4—C9	179.17 (14)	O3—S—C12—C13	159.04 (12)
C3—C4—C5—C6	−0.8 (2)	C1—S—C12—C13	−84.41 (13)
C9—C4—C5—C6	179.94 (15)	C17—C12—C13—C14	0.2 (2)
C4—C5—C6—C7	0.9 (2)	S—C12—C13—C14	−176.95 (12)
C4—C5—C6—C10	−179.56 (15)	C12—C13—C14—C15	0.7 (2)
C8—O1—C7—C6	−178.73 (14)	C13—C14—C15—F	179.52 (15)
C8—O1—C7—C2	0.24 (16)	C13—C14—C15—C16	−0.9 (3)
C5—C6—C7—O1	178.75 (13)	F—C15—C16—C17	179.80 (16)
C10—C6—C7—O1	−0.8 (2)	C14—C15—C16—C17	0.2 (3)
C5—C6—C7—C2	−0.1 (2)	C15—C16—C17—C12	0.7 (3)
C10—C6—C7—C2	−179.66 (14)	C13—C12—C17—C16	−0.9 (2)
C3—C2—C7—O1	−179.73 (12)	S—C12—C17—C16	176.28 (13)

### Hydrogen-bond geometry (Å, °)

**Table:**

*D*—H···*A*	*D*—H	H···*A*	*D*···*A*	*D*—H···*A*
C10—H10B···O2^i^	0.98	2.56	3.363 (2)	139
C13—H13···O2^ii^	0.95	2.45	3.365 (2)	160
C17—H17···O3^iii^	0.95	2.50	3.198 (2)	130

Symmetry codes: (i) −*x*+1, −*y*+1, −*z*+1; (ii) −*x*+1, −*y*+2, −*z*+1; (iii) −*x*+1, −*y*+2, −*z*.
